# Procedure using CAD/CAM-manufactured insertion guides for purely mini-implant-borne rapid maxillary expanders

**DOI:** 10.1007/s00056-022-00375-w

**Published:** 2022-02-21

**Authors:** Benedict Wilmes, Nour Eldin Tarraf, Renzo de Gabriele, Gianluca Dallatana, Dieter Drescher

**Affiliations:** 1 Department of Orthodontics, University of Duesseldorf, Moorenstr. 5, 40225 Duesseldorf, Germany; 2 Private Specialist Orthodontic Practice, Sydney, Australia; 3 Private Specialist Orthodontic Practice, Lecce, Italy; 4 Dental technician, Parma, Italy

**Keywords:** Rapid maxillary expansion, Temporary anchorage device, Skeletal anchorage, Mini-implants, Quadexpander, Gaumennahterweiterung, Temporary Anchorage Device, Skelettale Verankerung, Mini-Implantate, Quadexpander

## Abstract

With traditional rapid palatal expansion (RPE), orthopaedic forces are transmitted to the skeletal structures via the anchor teeth potentially leading to several unwanted dental side effects. To prevent these issues, tooth–bone-borne or purely bone-borne expanders were introduced using mini-implants in the palate. In this paper, the digitally planned Quadexpander is described which permits palatal expansion with only skeletal anchorage. The use of virtual insertion planning allows for insertion in areas of ideal bone, while avoiding roots and vital structures as well as the possibility of insertion into sites which would otherwise not be considered usable. A second advantage of digital planning is that mini-implants and the expander can be inserted in just one appointment.

## Abstract (de)

Bei der herkömmlichen Gaumennahterweiterung (GNE) werden orthopädische Kräfte über Zähne auf die skelettalen Strukturen übertragen, was zu einer Reihe unerwünschter dentaler Nebenwirkungen führen kann. Um diese Problematik zu verhindern, wurden Zahn-Knochen-gestützte oder ausschließlich knochengestützte Expander unter Verwendung von Mini-Implantaten im Gaumen eingeführt. In diesem Beitrag wird der digital geplante Quadexpander vorgestellt, der eine palatinale Expansion mit ausschließlich skelettaler Verankerung ermöglicht. Die virtuelle Insertionsplanung ermöglicht die Insertion in Regionen mit idealem Knochenvolumen, wobei von Zahnwurzeln und anderen wesentlichen Strukturen Abstand gehalten wird. Zudem bietet sie die Möglichkeit der Insertion in Regionen, die sonst nicht als geeignet ersichtlich wären. Ein zweiter Vorteil der digitalen Planung besteht darin, dass die Mini-Implantate und der Expander in einem einzigen Termin eingesetzt werden können.

## Introduction

Rapid palatal expansion (RPE) was first described by Angell [1] in 1860 and has been widely used to skeletally expand the maxilla. The orthopaedic forces are transmitted to the skeletal structures via the anchor teeth, which may lead to several unwanted dental side effects such as buccal tipping, fenestration of the buccal bone, root resorption, and gingival recessions even in children [2, 5, 7, 25]. In adults, resistance to expansion is too high and osteotomies were advocated to facilitate skeletal expansion and reduce the stress on the anchor teeth. However the dental side effects are reduced but not eliminated [8, 22]. To overcome these drawbacks, Mommaerts proposed the purely bone-borne transpalatal distractor (TPD) [9, 14, 19]. However, insertion and removal of these miniplate-borne distractors involves invasive surgical procedures with the need for flap preparation, risk of root injury and infections [9–11, 27].

In recent years, mini-implant supported RPE (MARPE, microimplant-assisted rapid palatal expansion) such as the Hybrid Hyrax [28, 29] and the MSE (maxillary skeletal expander [4, 18]) allow for the load of the expansion to be shared between two or four mini-implants in the palate and two first molars, greatly reducing the dental side effects of RPE and early class III maxillary protraction [20, 21, 30, 31] with minimally invasive placement procedures [3]. However, when expansion is desired in mature individuals it is preferable to avoid any loading of the dentition as the loads transmitted to the teeth would be extreme in case of undetected mini-implant failure during expansion.

Mini-implants have a high success rate in the anterior palate when inserted in the so-called T‑zone [12, 17, 32], the area of the bicuspids median or paramedian. This area provides good bone quality and minimal risk of injury to nerves, vessels or roots. However, further posteriorly in the molar area, bone availability is limited paramedian to the suture and mini-implants may be inserted palatally in the alveolar process between the first molar and second premolar [13, 17, 32]. In this area, safe insertion without root damage is crucial. The use of a mini-implant placement guide [6, 33], akin to those used in the placement of dental implants, would significantly aid risk-free mini-implant placement as well as allow the prefabrication of the pure bone-borne expander with four mini-implants (Quadexpander).

## Materials and methods

### Virtual planning of the mini-implant position and manufacturing of a Quadexpander

#### Step 1:

An STL file of the upper jaw is obtained either via an intraoral scan or a scan of a study model produced using a high-quality silicon impression. The surface mesh of the upper jaw is superimposed with a cone beam computed tomography image to identify an optimal site for mini-implant placement. The superimposition is performed using proprietary software (Easy Driver V 2.0.2019, Uniontech, Parma, Italy) in a two-step process, whereby firstly three common points between the CBCT (Cone beam computed tomography) and the surface mesh are identified and then the cross sections of the digital model and CBCT are matched to produce an accurate superimposition.

#### Step 2:

The virtual planning software is then used to confirm the precise anatomical positioning of the TADs. The employed software allows for the virtual planning and placement of TADs in a variety of lengths and diameters and for the ideal positioning of the screws according to the anatomical variations of each patient. The CBCT allows a three-dimensional orientation to select an area with optimal bone quantity and quality. Using different filters, it is possible to set the level of transparency and to clearly determine the ideal TAD placement.

#### Step 3:

Once the position of the mini-implants is finalized, a 3D printed model is created from the virtual model with the planned mini-implant positions represented by laboratory implant analogues. These steps are done using the software Easy Driver V 2.0.2019 (Uniontech, Parma, Italy, patent protected). The analogues are inserted manually in the marked locations in a 3D printed model or transferred to a plaster model in the orthodontic laboratory. The Quadexpander is then manufactured on this model using a preformed screw (Power Expander, Tiger Dental, Bregenz, Austria) welded to four preformed rings (PSM, Gunningen, Germany) which are positioned on the laboratory TAD analogues and connected to the expansion screw while bending the arms and adapting it to the shape of the palate.

#### Step 4:

The insertion guide is virtually designed around the mini-implant positions and then printed from a biocompatible resin VeroGlaze Med 620 (Seido-Systems, Kortrijk, Belgium) using a 3D printer (Stratasys LTD, Eden Prairie, MN, USA). Intraorally, this guide allows a precise location of the mini-implants. Removable sleeves in the shape of precise cylinders (peek material, brown color) are additionally employed to ensure precise coupling with the mini-implant driver for accurate insertion.

#### Step 5:

Mini-implants with an inner thread (2.3 mm diameter, Benefit, PSM, Gunningen, Germany) are inserted through the surgical guide using a contra-angle screwdriver. A special mini-implant insertions kit including the removable sleeves is used, which is designed to precisely fit into the insertion guide cylinders to ensure correct transfer of the planned mini-implant position very similar to what is used with dental implant placement guides.

#### Step 6:

At the same appointment, the prefabricated Quadexpander is fitted to the four mini-implants using four fixation screws (Benefit system, PSM, Gunningen, Germany) and expansion can commence.

### Clinical example 1

**Fig. 1:**
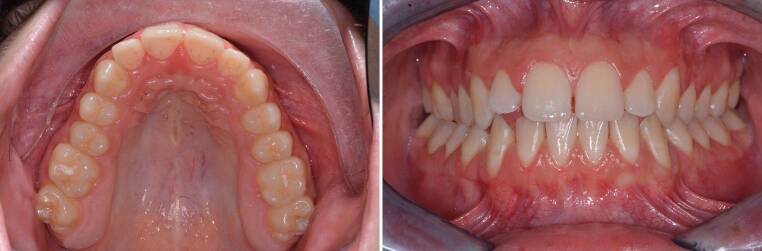
Transversal maxillary deficiency of an 18-year-old patient (case 1) Transversale maxilläre Hypoplasie bei einem 18-jährigen Patienten (Fall 1)

**Fig. 2:**
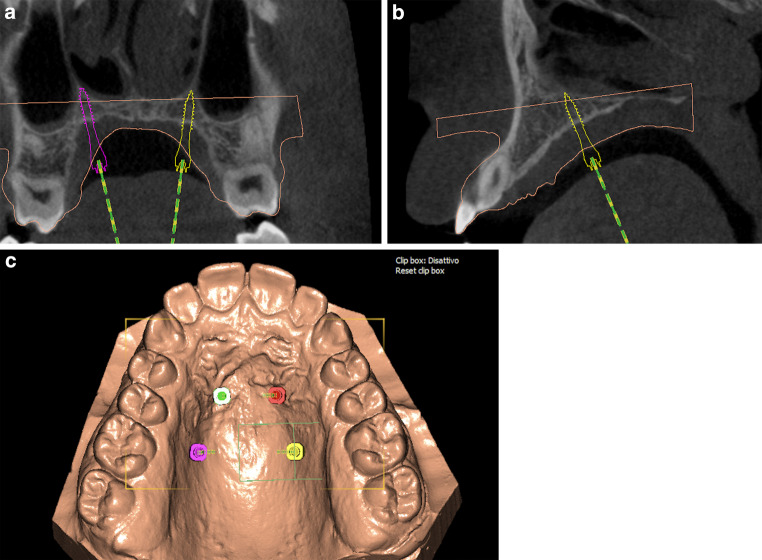
After superimposition of the digitized model and the cone-beam computed tomographic (CBCT) image, the optimal anterior and posterior positions of the mini-implants are virtually planned (Easy Driver V 2.0.2019, Uniontech, Parma, Italy) (case 1) Nach der Überlagerung des digitalisierten Modells und der DVT-Aufnahme wird die optimale anteriore und posteriore Position der Mini-Implantate virtuell geplant (Easy Driver V 2.0.2019, Uniontech, Parma, Italien) (Fall 1)

**Fig. 3:**
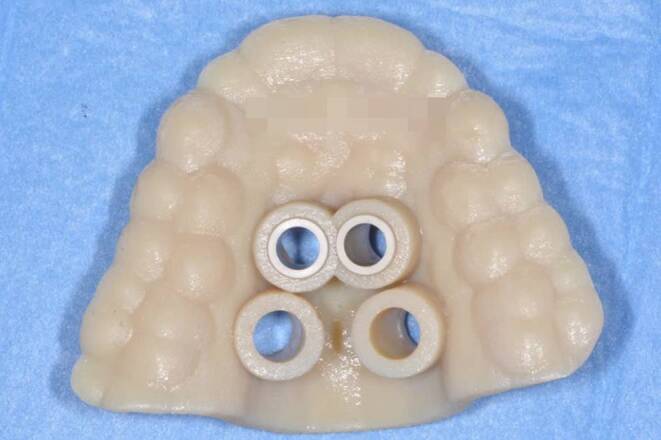
Insertion guide (Seido-Systems, Kortrijk, Belgium; Stratasys LTD, Eden Prairie, MN, USA) for case 1: insertion site, depth and angulation are predetermined Insertionsguide (Seido-Systems, Kortrijk, Belgien; Stratasys LTD, Eden Prairie, MN, USA) für Fall 1: Insertionsregion, -tiefe und -winkel sind vorgegeben

**Fig. 4:**
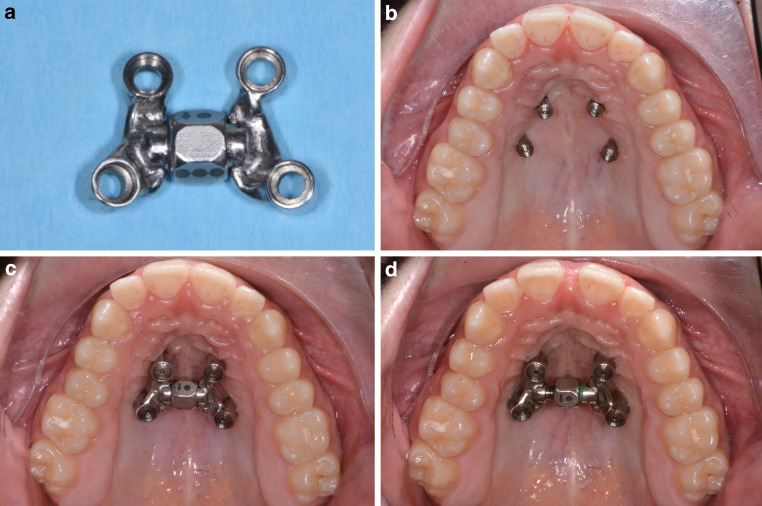
Clinical procedure of case 1: **a **Quadexpander with a Power Screw (Tiger Dental, Bregenz, Austria), **b** temporary anchorage devices (Benefit System, PSM, Gunningen, Germany) after insertion. **c,d **Before and after expansion Klinischer Verlauf, Fall 1: **a **Quadexpander mit einer PowerScrew (Tiger Dental, Bregenz, Österreich), **b **temporäre Verankerungen (Benefit System, PSM, Gunningen, Deutschland) nach dem Einsetzen. **c,d **Vor und nach Erweiterung

An 18-year-old male patient presented with a transverse maxillary deficiency due a narrow maxilla (Fig. 1). Because of the relatively mature age of the patient for conventional palatal expansion and buccal recessions, a purely bone-borne expansion appliance was chosen using orthodontic mini-implants. For the described planning procedure, a CBCT was obtained and superimposed with the digital model. The anterior and posterior optimal positions of the mini-implants were digitally planned (Fig. 2). Subsequently, the insertion guides were produced by the described computer-aided design/computer-aided manufacturing (CAD/CAM) procedure (Fig. 3). The mini-implants and the Quadexpander (with a “Power expander”, Tiger Dental, Bregenz, Austria) were inserted in the same session (Fig. 4). The patient was instructed to turn the expander twice per day resulting in a daily activation of 0.34 mm per day (2 × 0.17 mm). After 30 days, sufficient expansion was achieved (Fig. 4).

### Clinical example 2

**Fig. 5:**
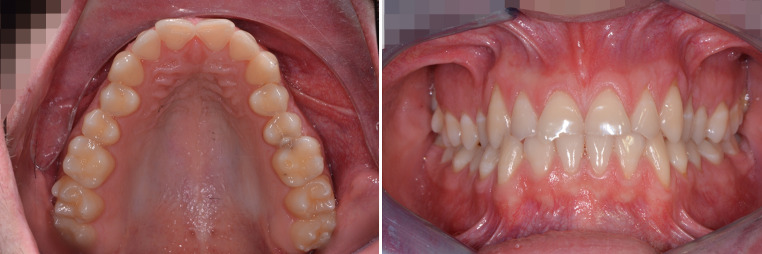
Transversal maxillary deficiency in a 22-year-old patient (case 2) Transversale maxilläre Hypoplasie bei einem 22-jährigen Patienten (Fall 2)

**Fig. 6:**
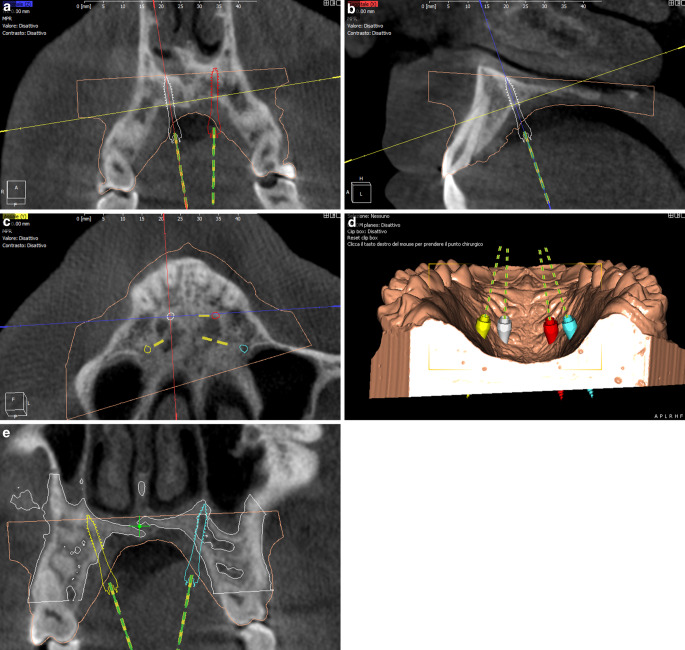
Virtual planning (Easy Driver V 2.0.2019, Uniontech, Parma, Italy) of optimal insertion sites for palatal temporary anchorage devices in the anterior (**a–d**) and posterior (**c**–**e**) palate (case 2) Virtuelle Planung (Easy Driver V 2.0.2019, Uniontech, Parma, Italien) der optimalen Insertionsstellen für die temporären palatinalen Verankerungen im anterioren (**a–d**) und posterioren (**e**) Gaumen (Fall 2)

**Fig. 7:**
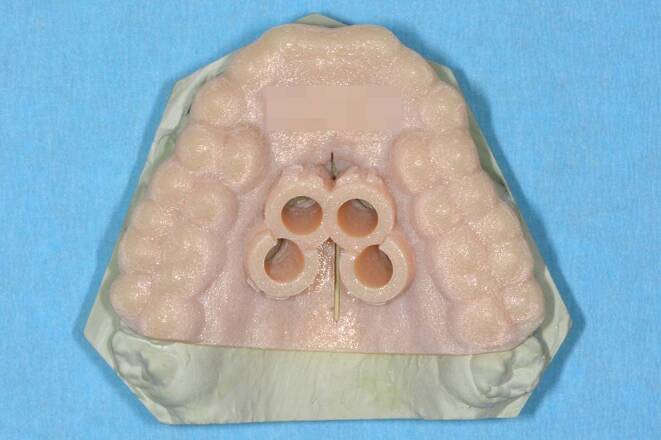
Insertion guide (Seido-Systems, Kortrijk, Belgium; Stratasys LTD, Eden Prairie, MN, USA) for case 2: insertion site, depth and angulation are predetermined Insertionsguide (Seido-Systems, Kortrijk, Belgien; Stratasys LTD, Eden Prairie, MN, USA) für Fall 2: Insertionsstelle, -tiefe und -winkel sind vorgegeben

**Fig. 8:**
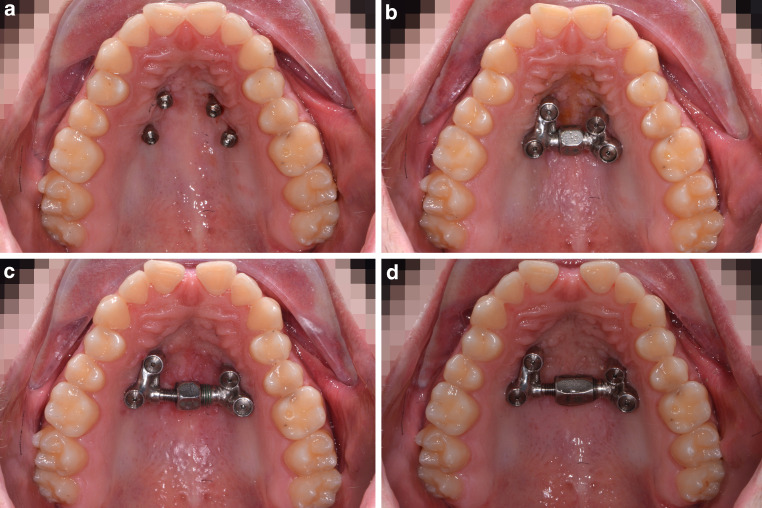
Clinical follow-ups of case 2 (22-year-old man), after insertion of four palatal temporary anchorage devices (**a**), expander in situ (**b**), after expansion with the first nut (**c**), after continuing of the expansion with the bigger nut (**d**) Klinische Nachuntersuchungen von Fall 2 (22-jähriger Mann), nach Einsetzen von 4 temporären palatinalen Verankerungen (**a**), Expander in situ (**b**), nach Expansion mit der ersten Nut (**c**), nach Fortsetzung der Expansion mit der größeren Nut (**d**)

A 22-year-old male patient presented with a narrow maxilla and buccal recessions (Fig. 5). A Quadexpander with 4 mini-implants was chosen to perform RPE without invasive surgery. The CBCT was superimposed with the digital model for the virtual positioning of the mini-implants (Fig. 6). Subsequently, the insertion guides were produced (Fig. 7). The mini-implants and the Quadexpander were inserted in the same session (Fig. 8). The patient was instructed to turn the expander twice per day resulting in a daily activation of 0.34 mm per day (2 × 0.17 mm). When the maximum capacity of the expander was reached, the nut was exchanged chairside to continue the expansion. After 32 days, a sufficient expansion was achieved (Fig. 8).

## Discussion

When attempting rapid maxillary expansion in an adult patient, the resistance to expansion is substantially increased. With the advent of mini-implants, a minimally invasive expansion has been introduced in older teenagers and adults (MARPE). The Quadexpander is a purely bone-borne mini-implant supported maxillary expansion appliance. This eliminates the risk of the anchorage teeth being damaged in case of undetected mini-implant failure. Due to the very thin cortical bone support in the paramedian areas of the posterior palate this insertion area was dismissed. Even though this is an area where MSE [4, 18] screws are placed, the MSE appliance shares the load of expansion with the posterior teeth and it is difficult to estimate the real contribution of the posterior mini-implants in actual load bearing and how much of that load is transmitted to the molars. The Quadexpander eliminates this risk by being purely bone-borne. Success of *all* the mini-implants is crucial when the expander is purely implant-borne as not only will the mini-implants bear the load during the expansion phase but also during the retention period which is likely to be several months for an adult. As a consequence, to avoid any risk of root proximity or injury, insertion guides are proposed for the TAD placement. Insertion guides are now widely used in the placement of prosthetic dental implants [26]. Static computer aided guided surgery (s-CAIS), static implant guides are the most commonly used type [23]. The clinician can gain insight into the placement site, bone quality as well as any anatomical variations. This pre-operative planning allows the clinician to have some peace of mind during the procedure and he or she can focus more on tissue handling and patient management. Most of the guides used in prosthodontic implants use specifically designed implant placement kits [24], which couple precisely with the drill and placement guides the equivalent of which was not available with orthodontic mini-implant kits. In fact, to date such guides have not been widely used with orthodontic mini-implant placement although there have been several attempts using prebent wire guides in combination with conventional mini-implant placement tools, which do not provide a great degree of precision. The Easy Driver system, however, provides a specifically designed mini-implant placement kit designed to fit the placement guides with very little tolerance, akin to those used with dental implants to allow precise insertion of the mini-implants into the planned position. By preplanning the position of the implant placement, the area with the best bone can be selected. In addition, using the CBCT as a guide, allows for the use of an implant that not only engages the palatal cortical plate but also the floor of the nose allowing for bicortical engagement, further increasing the primary stability [16] and success of the TADs. Furthermore, the use of a precise placement guide could allow for safe insertion of TADs in the presence of palatally impacted canines or unerupted teeth discovered in late teen or young adults. Studies have also shown that proximity of TADs to tooth roots can predispose TAD failure [15]. The diameter of the TAD can then be varied as well. Several types of expanders have been proposed in the literature using TADs. The described 4‑screw expander (Quadexpander) permits safe and easy insertion of the TADs even by orthodontists without much experience in mini-implant insertion. The mini-implant and expander insertion can be done in one clinical session saving significant chair time. Compared to the MSE [4, 18] appliances employment of insertion guides allows high flexibility in the placement of the TADs to ensure all four mini-implants are placed in the best quality bone as opposed being restricted by the fact the screws with MSE must follow the outline of the prefabricated expansion screw which may place one or more screws in an area with lesser bone quality.

The Quadexpander can be used in adolescents and adults, as well as in patients with missing teeth. Since teeth are not needed as anchorage units, this approach might be interesting especially for patients with periodontically compromised teeth, e.g. buccal recessions. Furthermore, it is possible to use the 4‑screw expander in conjunction with a surgically assisted RME especially in periodontally compromised patients, or if the expansion does not progress in older adults by performing a minimally invasive bilateral corticotomy. The fact that the appliance does not include any teeth means that tooth movement can be started independent of the expansion and the retention period required. This seems to be especially relevant if aligners should be used in the second phase of the orthodontic treatment [34]. A further advantage could be in cases where the orthodontist is not willing to place the TADs and the patient is referred to an oral surgeon. By providing the placement guides the orthodontist can be confident that the TADs will be in the desired locations.

The need for additional radiation exposure from a CBCT to manufacture the insertion guides is a disadvantage. However, in the selected cases where this type of expansion is required it can be justified. The following indications can be considered for the digitally planned Quadexpander:

- Minimally invasive skeletal maxillary expansion in older adolescents and adults.
- Maxillary expansion in cases with multiple missing teeth.
- Maxillary expansion in the presence of a periodontally compromised dentition, e.g. when buccal recessions are diagnosed.
- Safe placement of mini-implants for expansion in cases with palatally impacted teeth.

## Conclusion

To overcome issues of tooth-borne expanders in older adolescents and adults, digitally planned Quadexpanders can be employed which permit palatal expansion with only skeletal anchorage. The use of virtual insertion planning allows for insertion in areas of ideal bone while avoiding root damage. A second advantage of digital planning is that mini-implants and the expander can be inserted in just one appointment.
